# Community perceptions of epilepsy and its treatment in an onchocerciasis endemic region in Ituri, Democratic Republic of Congo

**DOI:** 10.1186/s40249-018-0498-0

**Published:** 2018-12-04

**Authors:** Housseini Dolo, Michel Mandro, Deogratias Wonya’Rossi, Francoise Ngave, Jessica Fraeyman, Joseph N. Siewe, Patrick Suykerbuyk, Robert Colebunders

**Affiliations:** 1 Global Health Institute, University of Antwerp, Antwerp, Belgium; 2 Provincial Ministry of Health, Bunia, Ituri Democratic Republic of the Congo; 3 International Center of Excellence in Research, Faculty of Medicine and Odonto Stomatology, Bamako, Mali; 4 Research Group Social Epidemiology and Health Policy, University of Antwerp, Antwerp, Belgium; 5 Centre de Recherche en Maladies Tropicales de l’Ituri, Hopital General de Reference de Rethy, Rethy, Democratic Republic of Congo

**Keywords:** Epilepsy, Onchocerciasis, Community, Perception, Experiences, Treatment, Programme, Qualitative study

## Abstract

**Background:**

A recent study in the Logo and Rethy health zones in the Ituri Province in the Democratic Republic of Congo (DRC) reported that the majority of the persons with epilepsy (PWE) had not been treated with anti-epileptic medication (AEM) or had stopped treatment. Prior to the implementation of an epilepsy treatment programme in these health zones, this study investigated the perceptions and experiences regarding epilepsy and its treatment amongst community leaders, PWE and/or their families, traditional healers and health professionals.

**Methods:**

A total of 14 focus group discussions (FGD) and 39 semi-structured interviews (SSI) were conducted with PWE and/or their family members, community leaders, traditional healers, and health professionals in the Logo and Rethy health zones during February 2–19, 2017.

**Results:**

In the two health zones, the clinical signs of convulsive epilepsy were recognized by community members. However, a variety of misconceptions about epilepsy were identified including the beliefs that epilepsy is a family-related condition, a contagious disease, is transmitted by insects, saliva or by touching a person of the same sex during seizures, or is caused by evil spirits and witchcraft. The role of traditional healers in spreading these beliefs was revealed. The study also reported sexual abuse on PWE, stigmatisation of PWE and loss of productivity of PWE and their families. Some PWE had been using AEM and traditional treatment but were not convinced about the efficacy of these treatment options. The lack of training of health providers about epilepsy care, financial barriers in obtaining AEM, and the shortage of AEM at primary health facilities were revealed. As remedies, the community requested access to a decentralized center for epilepsy treatment. They also proposed using churches and community health workers as communication channels for information about epilepsy.

**Conclusions:**

Clinical signs of convulsive epilepsy were recognized by the community in the Logo and Rethy health zones but many misconceptions about epilepsy were identified. A comprehensive community-based epilepsy treatment programme with an affordable uninterrupted AEM supply needs to be established. Such a programme should address stigma, misconceptions, sexual abuse and foster the rehabilitation of PWE to alleviate poverty.

## Background

Epilepsy is a major public health problem affecting about 50 million people worldwide, 80% of whom live in resource-limited countries [1]. The prevalence of epilepsy in Sub-Saharan Africa and Latin America is particularly high, with respectively, 15 and 18 per 1000 people affected, compared to 6 per 1000 people in Asia, and less than 8 per 1000 people in Europe and Northern America [2]. Epilepsy is associated with important medical, psychological, economic and social consequences for the affected individuals and their families as well as their communities. Indeed, the socio-cultural implications of epilepsy including taboo, stigma and exclusion are well known [3, 4]. Moreover, persons with epilepsy (PWE) are confronted with socio-cultural and health barriers, resulting in insufficient access to treatment, especially anti-epileptic medications (AEM) [5, 6].

Epidemiological studies conducted in onchocerciasis endemic areas in Central and Eastern Africa have documented an association between onchocerciasis and epilepsy prevalences [7–9]. Recently, in certain onchocerciasis endemic villages in the Logo and Rethy health zones in Ituri in the Democratic Republic of Congo (DRC) an epilepsy prevalence between 3.6–6.2% was reported [7]. Another study in these same health zones revealed that the majority of the persons with epilepsy (PWE) had never been treated with AEM or had stopped treatment [10]. Therefore, prior to the implementation of an epilepsy treatment programme in these health zones, we investigated the perceptions and experiences with epilepsy and its treatment amongst community leaders, PWE and/or their families, traditional healers and health professionals to determine how such programme should be set up.

## Methods

### Study sites

The villages of Ndroy, Kpana and Kanga in the Logo health zone and the villages of Lokpa, Kpagboma and Rassia in the Rethy health zone were selected for this study. All these villages are located in an onchocerciasis endemic region in the Ituri Province [7]. Both health zones are post conflict regions of the DRC with weak health systems. At the time of the study, the Logo and Rethy health zones had an estimated population of 255 485 and 218 807 inhabitants, respectively [11]. Alur and *Bbaledha* are the dominant languages in the Logo and Rethy health zones, respectively. Swahili is the common language for communication in the two health zones.

### Study design

A total of 14 focus group discussions (FGD) and 39 semi-structured interviews (SSI) were conducted with PWE and/or their family members, community leaders, traditional healers, and health professionals.

### Study participants and sampling procedure

Firstly, information and awareness-raising visits were carried out in villages together with the heads of the Jukoth and Zabu communities in the Logo and Rethy health zones respectively. The involvement of community heads during these visits facilitated access to both the general population and traditional healers. Secondly, a series of meetings were held to explain the study objectives and methodology to the medical officers of the Logo and Rethy health zones in order to obtain their authorizations to visit the health facilities. In each facility, we explained the study protocol to the nurses in charge.

Community leaders, PWE and/or their family members, traditional healers and health professionals were informed one day before the research team’s visit to each village or health center. The constitution of FGD was done purposively, according to each participant’s availability and willingness to participate. FGD were conducted separately with community leaders, PWE and/or their family members and traditional healers in each selected village. Each FGD contained between 6 and 10 participants. All known traditional healers in each health area were invited to participate. Semi-structured interviews (SSI) were conducted with PWE and /or their family members, and health professionals. In each health center, the principal nurse in charge was interviewed. One village in the Rethy health zone was chosen to pretest interview guides containing discussion themes.

### Data collection

Data was collected from February 2–19, 2017. FGDs were conducted with community leaders, PWE and/or their families and traditional healers. SSI were conducted with PWE and/or their families members and health professionals. Participants were contacted one day before the interview to identify a suitable time frame to meet with the investigators.

A trained health professional (DWR), native of the study area and fluent in the local languages (*Alur*, *Swahili* and/or *Bbaledha*), acted as the moderator. At the end of each FGD and SSI, the moderator summarized the discussions in French to enable the principal investigator (PI) (HD) to ask additional questions. Both FGDs and SSIs were audio recorded.

### Data analysis

**Fig. 1:**
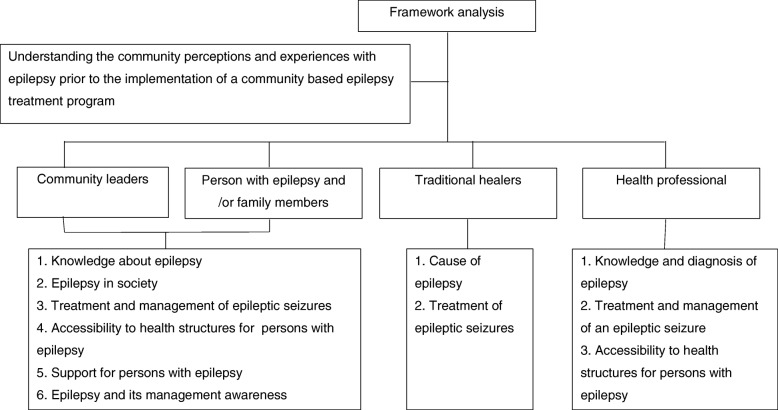
Research question with the framework analysis themes

Data from the FGD and SSI were transcribed by trained field staff, verbatim from the local languages to French. Subsequently a framework analysis was conducted as described by Gale et al. [12]. All data were coded and analysed according to the five stages of this method (familiarization, identification of a thematic framework, indexing, charting, mapping and interpretation). During the familiarization stage, the PI became familiar with the data by meticulously reading through transcripts on several occasions. For the identification of a thematic framework, the PI coded the first two transcripts for each target group before meeting with the rest of the team to discuss key themes and constructing an initial coding framework for each group. For indexing the data, the thematic framework was systematically applied to all transcripts using a qualitative data-analysis package (Quirkos® software version 1.5.0 https://www.quirkos.com/index.html). The PI provided codes to classify the opinions expressed during the different FGD and SSI in relation to the research question. (Fig. 1). To chart the data, a matrix was created for each theme by summarizing and charting data for each case and each code within that theme. To map and interpret the data, thematic analysis was carried out on the managed dataset by reviewing the matrices and making connections within and between codes and cases. This process was influenced by the original research questions as well as concepts generated inductively from the data as described by Pope et al. [13]. Quotes selected for the article were translated into English by the PI and other co-authors involved in the study.

## Results

A total of 14 FGD involving 60 community leaders, 35 PWE and/or their family members, six traditional healers and a total of 39 SSI with PWE and/or their family members, and health professionals were conducted.

We focused on seven main themes related to perceptions and experiences potentially important for the implementation of a community-based epilepsy treatment programme: “epilepsy related misconception”, “AEM and traditional treatment inefficacity”, “stigma and low socio-economic status of PWE and their families”, “community based epilepsy treatment”, “epilepsy awareness”, “AEM shortage and consultation fee as a barrier to obtain treatment”, “management of epilepsy and need for health staff capacity building”. The identified experiences and perceptions by different target groups were reported as follows (Table 1).

**Table 1:** Summary of main result by themes and by target group

Themes and issues	Community leaders	PWE /families	Traditional healers	Health professional
Misconceptions concerning the cause of epilepsy	✓	✓	✓	
Epilepsy is a family-related condition		✓	✓	
Epilepsy is a contagious disease	✓			
Epilepsy is caused by bad spirits, witchcraft	✓	✓	✓	
Stigma	✓	✓		
Epilepsy is associated with stigma within families and communities	✓	✓		
Epilepsy leads to divorce or separation	✓	✓		
PWE are at risk for sexual abuse	✓	✓		
Epilepsy treatment	✓	✓	✓	✓
Not convinced about efficacy of traditional treatment and AEM	✓	✓		
Need to establish a community-based specialized epilepsy treatment center	✓			
Financial possibilities limit the uninterrupted intake of AEM	✓	✓		
Consultation fee is barrier to obtain AEM	✓	✓		✓
AEM are often not available	✓	✓		✓
Need to train health professionals about the management of epilepsy	✓			✓
Epilepsy awareness	✓	✓		
Clinical signs of convulsive epilepsy were recognized	✓	✓		
Economic status of PWE/Families	✓	✓		
Low economic status of PWE and their families	✓	✓		
PWE are dependent on their families to obtain treatment and care	✓	✓		

*PWE* Person with epilepsy, *AEM* Anti-epileptic medication

### Community leaders

#### Epilepsy related misconceptions

During the FGD, the beliefs that epilepsy is a contagious disease transmitted by insects and/or saliva, and/or by touching a person of the same sex during seizures were expressed by several community leaders.

“*During the seizures we should not touch the PWE, or that men should not hold men who are having a seizure, or that women should not hold a woman having a seizure*.” [community leaders FGD 1].

Most community leaders were skeptical about the efficacy of AEM and they considered epilepsy as an incurable condition.

“*The medicine against epilepsy, is there any that has been discovered to date? When we carry PWE to traditional healers, we are deceived because the seizures do not stop. If you have the drugs bring them to us”*. [community leaders FGD 3].

#### Stigma

Stigmatization of PWE occurs within families as well as in communities. In the community, PWE are labeled as individuals who are mentally ill and therefore are not allowed to live like other members of the community.

“*There are some persons who see PWE as shameful. There are others who do not like to stay with these sick people, they are ashamed to stay with a PWE and they say that PWE dishonor them, others do not even allow them to be seen, they hide PWE, when visitors arrive, they say: ‘these dirty guys should be far away’* ”. [community leaders FGD 1].

#### Epilepsy treatment

Despite the fact that PWE seek care using modern and traditional treatment, the community leaders are not convinced about the capacity of these two options to cure epilepsy. However, they mentioned that AEM are capable of reducing the intensities and frequencies of seizures.

“*When a PWE takes the medicine, he does not suffer from seizures but as soon as there is an interruption of the drug the seizures will resume*” . [Community leaders FGD 5].

“*For this disease[epilepsy], there are many problems associated with its treatment. We travelled to traditional healers for treatment, it does not help, also the drugs we bought in pharmacies do not help…*” [Community leaders FGD 5].

Community leaders proposed the establishment of a community-based epilepsy treatment center and/or bringing specialized staff into the village or its vicinity for effective management of epilepsy and associated morbidities including burns and injuries due to seizures.

*“I think that, to solve this problem of epilepsy, we have to design and build a structure of care specifically dedicated for PWE. If we could have a specialized trained staff, who understands epilepsy and its treatment, let him come and stay with us in this village.”* [community leaders FGD 1].

Community leaders suggested informing the population about epilepsy using churches, traditional chiefs and community health workers.

“*One could say this (information about epilepsy) through the churches*”. [community leaders FGD 1].

“*The community health workers also could serve as information channel for epilepsy*”. [community leaders FGD 1].

### PWE and their families

#### Epilepsy awareness

Epilepsy is known as “*Timbu*” in the Logo and Rethy health zones. Nonetheless, the participants in the different FGD and SSI sessions never pronounced the word *“Timbu”.* They rather preferred to use the term “condition” or “thing” or “seizure”. The majority of participants were able to describe epilepsy characterized by tonic-clonic seizures.

*“*…*.. this thing (epilepsy) arrives suddenly…. you will be surprised that the seizures have made him fall”.* [PWE/families FGD 2].

“*That thing (epilepsy), when it has made people fall, ... to bring them for care is difficult since they convulse with force. … and finally, there body will calm down and then they regain consciousness, they have already wounded their tongues”*. [PWE/families FGD3].

#### Epilepsy related misconceptions

Many people believed that epilepsy is a family-related condition.

“*Since I (woman) got married, it is the first child who has this disease among my three children. I was told that in his family (of the husband) this disease is present. But in the family of other children (healthy children) there is no PWE and they do not have this disease*”. [PWE/families SSI 6.18].

PWE also believed that there is no cure for epilepsy.

“*We have not seen anyone cured by these drugs; they reduce the intensity of the disease, but the seizure resumes after sometime. We bought drugs a lot but curing epilepsy, we never observed that.”* [PWE/families FGD 2].

“*If someone says he can cure epilepsy it is false*! *”.* [PWE/families SSI 3.15].

#### Stigma

In some families, the PWE are rejected or not considered as family members during social events and celebrations.

“*People look at the PWE with disrespect, and they speak with disregard. They say that the child is crazy. I have been told myself that my child is a mad man, I must not let him walk among people. While this disease makes PWE walk here and there like crazy.”* [PWE/families FGD 3].

PWE can find a partner to marry but once the epilepsy is disclosed the PWE will lose her/his partner. In couples where one partner has epilepsy, this may lead to divorce or separation. Moreover, PWE are subject to sexual abuse.

*“She [PWE] already got married once. Since she is weak, sometimes she is not able to do small business to get money, therefore, I had removed her from her home (husband’s home), because it is the woman who takes care of family’s expenses and she is not able to do so. I removed her to stay and suffer with me”.* [PWE/families SSI 5.22].

“*With their [PWE] abnormal intelligence, they can get married. But you as a parent will be worried at any time to know how they live in their husbands’ homes*” *.* [PWE/families SSI 3.9].

*“Like this pregnant teenager with epilepsy there! she does not even know from whom she got pregnant”.* [PWE/families FGD 6].

#### Economic status of PWE and their families

The productivity and ability to work is limited among the PWE and their families. They are seen as handicapped. It was also reported that a PWE is very dependent on his/her family such that his/her caregivers, generally the parents, no longer have the possibility to work because they must continually care for the PWE. This will increase the poverty of the family.

“*While you are working in the field, after working not much time in the farm, you are followed by somebody to tell you that your child has seizures. So, in this condition the way of finding money is too difficult; but you cannot give up your own child*”. [PWE/families FGD 2].

#### Epilepsy treatment

Epilepsy is treated using AEM and traditional treatment using sprays (in the churches), plants (mainly one plant called “*Dodoi*”), incantations (to chase away evil spirits) and bathing in the river. Health seeking behaviors of families depend on their financial possibilities and their experiences with the efficacy of the chosen treatment option. However, people from the Christian religion did not accept traditional treatment as “this could put them in conflict with their religious beliefs”.

“*Since I was sick, they wanted me to use the indigenous treatment: TYE ND JOK (a kind of veneration, sacrifice to the spirits of the Alur [tribe in DRC] ancestors). I refused that, I said it cannot work for me, because it will block me from going to the heaven. After that, I went to Logo hospital to get modern medicine*” . [PWE/families SSI 5.2].

*“When seizures are there, we will try to protect him first until he calms down. If there are drugs, they are given to him or we will try the plant [dodoy] in his nose”*. [PWE/families SSI 2.20].

AEM are often not available at the community health center and many participants reported buying AEM from private pharmacies. The most common reason for not going to the health center to obtain AEM is financial limitations. Parents/caregivers of PWE mentioned the chronic nature of epilepsy and the dependence of the PWE on other family members, which drives the family into poverty. The issue of a consultation fee that must be paid at the health center was also raised as a hinderance by both PWE and health professionals.

“*The medication for PWE does not exist at the health center here. However, health professionals would like that we give them money so that they bring us the medicines back from Logo [major township in the area] but we refused to give them money*” . [PWE/families SSI 6.12].

“*Yes. Sometimes the AEM are missing at the health center, and we go to pharmacies where we can find them*”. [PWE/families SSI 5.22].

“*It is very expensive. We asked a pharmacist to bring us a box of phenobarbital, it costs 60,000 shillings (close to US $ 20)”.* [PWE/families SSI 5.11].

“*The cost for AEM is high. When you give 1,000 shillings (about US $ 35 cents), you get only 5 tablets of phenobarbital*” [PWE/families SSI 6.12].

### Traditional healers

#### Epilepsy related misconceptions

The traditional healers considered epilepsy as a contagious disease, transmitted by insects, saliva, and by touching a person of the same sex during seizures. Concerning the cause of epilepsy, the majority of traditional healers believed that epilepsy is due to a spirit, witchcraft or an unknown cause.

“*During seizure, a man should be assisted by a woman, if another man does so, he will get epilepsy*”. [Traditional healers, FGD 1].

“*The epilepsy I am talking about is actually of two categories …, in many cases, it is discovered that it is the spirit……. Second category, comes from some families who have magic powers that cause epilepsy, such as the chalice of witchcraft.”* [Traditional healers, FGD 1].

“*We know each what gives epilepsy disease for the first time, it’s the small insects, which are found in lowlands”.* [Traditional healers, FGD 2].

#### Health professionals

##### Epilepsy treatment

The health professionals agreed that AEM shortage is an issue but that recently there is increased access.

“*AEM shortage can occur; but currently with the “CAMENIHU” (Centrale d’Achat et de distribution des Medicament Essentiels au Nord de l’Ituri et au Haut –Uele) there is no shortage”.* [Health professional SSI 1].

Health professionals stressed the need for training in the management of epilepsy. The three nurses interviewed had difficulties answering questions about how to diagnose epilepsy and to explaining the causes of epilepsy. They were also unaware of the relationship between epilepsy and onchocerciasis.

“*The epilepsy case management is problematic especially because clinical management is something we learned in school. But for the practice of clinical management, until now one does not master the correct prescriptions for AEM*” . [Health professional SSI 1].

## Discussion

This paper describes how communities in an onchocerciasis endemic area in Ituri with high epilepsy prevalence perceive and experience epilepsy and its treatment. The following challenges for the implementation of an epilepsy treatment programme were identified.

Many misconceptions about epilepsy were reported during the FGD and SSI which might influence the uptake of AEM by PWE. These misconceptions are the consequence of a lack of health education and training of local health professionals about epilepsy. Misconceptions such as epilepsy is a family-related condition, is a contagious disease were also reported in other studies in Africa [3, 4]. These beliefs were reported to be spread by traditional healers and shared with community members. Consequently, people put these beliefs into practice in their relationship with PWE and their families thus increasing the stigmatization of those affected. However, in a study conducted in Cameroon, although some negative practices of traditional healers were reported, it was also noted that traditional healers had a more positive attitude toward PWE in the Batibo health district compared to the general public in this community [14]. Therefore, a community-based misconception mitigation strategy needs to be developed by working closely with traditional healers.

An added advantage to addressing these misconceptions is the fact that increased awareness about epilepsy was shown to be associated with better self-management and adherence to epilepsy treatment [15]. Therefore, improving communities’ awareness about epilepsy should be a priority for the community based epilepsy treatment programme [16].

PWE and their families were reported to be stigmatized leading to social isolation and low socio-economic status. Consequently, PWE and their families have a tendency to boycott the public health structures to avoid being tagged as epileptic. Thus, the health condition of PWE may deteriorate due to lack of care and treatment as reported in Iran [17]. Moreover, sexual abuse of PWE was reported, as observed in other African countries [18, 19]. Health education campaigns, and peer support groups are required to decrease epilepsy-associated stigma and strengthen self-confidence and the personal values of PWE. Furthermore, awareness needs to be raised among politicians to legally protect PWE from sexual abuse.

Low socio-economic status hinders many PWE from obtaining epilepsy treatment. We discovered a double component to the poverty situation of PWE and their families: Firstly, the drugs are expensive and constitute a weight on the family budget; secondly, the seizures of PWE reduce the productivity of parents because they invest more time in taking care of affected family member instead of working in the farm. Financial difficulties were also found to be the main reason for non-adherence to AEM in the Mbam valley, Cameroon [20]. Moreover, AEM are not always available at the health centers and consultation fees also make access to epilepsy treatment difficult for many. Therefore, a community based epilepsy treatment programme should address the anti-epileptic drug shortage and consultation fee that still constitutes a barrier to obtaining epilepsy treatment. Without AEM, PWE are at risk of uncontrolled seizures and related complications including drowning, burns and traumatic lesions. Therefore, it is important to establish a decentralized system of treatment/care for PWE. Recently, a collaboration between the investigators involved in this study, the humanitarian organization Malteser International, and a drug supply agency named “CAMENIHU” (Centrale d’Achat et de distribution des Medicaments Essentiels au Nord de l’Ituri et au Haut –Uele) resulted in the implementation of a system to improve the drug supply and subsidize its cost. However, PWE still need to pay a consultation fee at the local health center, which is considered to be costly and not sustainable.

Most PWE are dependent on their families to obtain treatment and care, which in turn pushes them into poverty as reported in other resource limited settings [21, 22]. Therefore, a community based epilepsy treatment programme must try to prevent or decrease financial dependence of PWE by exploring possibilities to engage them in low risk income-generating activities or fully subsidize the treatment of PWE. Many PWE, for financial reasons, only take AEM after recovering from a seizure episode. This strategy will rapidly lead to a new episode of seizures. Furthermore, irregular intake of AEM can aggravate seizures [23]. The importance of both an uninterrupted AEM supply at the local health centres and continuous intake of AEM needs to be underscored [24, 25].

The socio economic impact of epilepsy in Africa is considerable [26]. Meanwhile, it costs less than US $ 5 per year to treat a PWE [27]. Providing AEM free of charge is expected to have a major impact in reducing epilepsy-related morbidity and mortality, improve the quality of live and economic condition of PWE and their families. To reduce the cost for PWE even further, similar to HIV care, the number of health centre visits should be limited by training PWE and their families on self-management [28, 29]. In addition, task shifting of epilepsy management to specialized nurses and community health workers will be required [17]. Increasing access to AEM and improving treatment and care for PWE will decrease epilepsy-related stigma and will allow PWE to live normal lives with no or very few seizures.

Primary health care providers stressed the need for training in epilepsy management [18]. A telemedicine system should be considered to allow distance coaching of local health workers, as is currently being rolled out in other resources limited settings [30, 31] . In addition, periodic supervision visits to local health staff by a medical doctor trained in epilepsy should be considered in these endemic areas with a high prevalence of epilepsy. Churches and community health workers, are avaible for communication, sensitization and behavioral change strategies related to epilepsy.

The link between epilepsy and onchocerciasis was never mentioned during the FGD and SSI sessions. Recent studies suggest that regular ivermectin use may protect against onchocerciasis-associated epilepsy [32]. Therefore, community directed treatment of ivermectin (CDTI) should be implemented in the onchocerciasis endemic villages within the Logo health zone. In the villages of the Rethy health zone where CDTI is already implemented, explaining to the community that onchocerciasis may lead to epilepsy and that this form of epilepsy can be prevented by taking ivermectin, may motivate people to take this drug.

## Conclusions

Clinical signs of convulsive epilepsy were recognized by the community in the Logo and Rethy health zones but many misconceptions about epilepsy were identified. A decentralized, comprehensive community based epilepsy treatment programme with an affordable uninterrupted AEM supply needs to be established in these regions. Such a programme should include a community component that addresses stigma, misconceptions, sexual abuse and alleviates poverty. Establishing such a programme will require a major advocacy and lobbying effort to obtain sufficient funds to implement it.

## Abbreviations

AEM: Anti-epileptic medication
CAMENIHU: Centrale d’Achat et de distribution des Medicament Essentiels au Nord de l’Ituri et au Haut-Uele
FGD: Focus group discussion
PWE: Person with epilepsy
SSI: Semi structured interview
